# Computational optimization of associative learning experiments

**DOI:** 10.1371/journal.pcbi.1007593

**Published:** 2020-01-06

**Authors:** Filip Melinscak, Dominik R. Bach

**Affiliations:** 1 Computational Psychiatry Research, Department of Psychiatry, Psychotherapy, and Psychosomatics, University of Zurich, Zurich, Switzerland; 2 Neuroscience Center Zurich, University of Zurich, Zurich, Switzerland; 3 Wellcome Centre for Human Neuroimaging and Max Planck UCL Centre for Computational Psychiatry and Ageing Research, University College London, London, United Kingdom; Dartmouth College, UNITED STATES

## Abstract

With computational biology striving to provide more accurate theoretical accounts of biological systems, use of increasingly complex computational models seems inevitable. However, this trend engenders a challenge of optimal experimental design: due to the flexibility of complex models, it is difficult to intuitively design experiments that will efficiently expose differences between candidate models or allow accurate estimation of their parameters. This challenge is well exemplified in associative learning research. Associative learning theory has a rich tradition of computational modeling, resulting in a growing space of increasingly complex models, which in turn renders manual design of informative experiments difficult. Here we propose a novel method for computational optimization of associative learning experiments. We first formalize associative learning experiments using a low number of tunable design variables, to make optimization tractable. Next, we combine simulation-based Bayesian experimental design with Bayesian optimization to arrive at a flexible method of tuning design variables. Finally, we validate the proposed method through extensive simulations covering both the objectives of accurate parameter estimation and model selection. The validation results show that computationally optimized experimental designs have the potential to substantially improve upon manual designs drawn from the literature, even when prior information guiding the optimization is scarce. Computational optimization of experiments may help address recent concerns over reproducibility by increasing the expected utility of studies, and it may even incentivize practices such as study pre-registration, since optimization requires a pre-specified analysis plan. Moreover, design optimization has the potential not only to improve basic research in domains such as associative learning, but also to play an important role in translational research. For example, design of behavioral and physiological diagnostic tests in the nascent field of computational psychiatry could benefit from an optimization-based approach, similar to the one presented here.

## Author summary

To capture complex biological systems, computational biology harnesses accordingly complex models. The flexibility of such models allows them to better explain real-world data; however, this flexibility also creates a challenge in designing informative experiments. Because flexible models can, by definition, fit a variety of experimental outcomes, it is difficult to intuitively design experiments that will expose differences between such models, or allow their parameters to be estimated with accuracy. This challenge of experimental design is apparent in research on associative learning, where the tradition of modeling has produced a growing space of increasingly complex theories. Here, we propose to use computational optimization methods to design associative learning experiments. We first formalize associative learning experiments, making their optimization possible, and then we describe a Bayesian, simulation-based method of finding optimized experiments. In several simulated scenarios, we demonstrate that optimized experimental designs can substantially improve upon the utility of often-used canonical designs. Moreover, a similar approach could also be used in translational research; e.g., in the nascent field of computational psychiatry, designs of behavioral and physiological diagnostic tests could be computationally optimized.

## Introduction

A major goal of computational biology is to find accurate theoretical accounts of biological systems. Given the complexity of biological systems, accurately describing them and predicting their behavior will likely require correspondingly complex computational models. Although the flexibility of complex models provides them with potential to account for a diverse set of phenomena, this flexibility also engenders an accompanying challenge of designing informative experiments. Indeed, flexible models can often be difficult to distinguish one from another under a variety of experimental conditions [1], and the models’ parameters often cannot be estimated with high certainty [2]. Designing informative experiments entails formalizing them in terms of tunable design variables, and finding values for these variables that will allow accurate model selection and parameter estimation. This challenge is well exemplified—and yet unaddressed—in the field of associative learning research.

Associative learning is the ability of organisms to acquire knowledge about environmental contingencies between stimuli, responses, and outcomes. This form of learning may not only be crucial in explaining how animals learn to efficiently behave in their environments [3], but understanding associative learning also has considerable translational value. Threat conditioning (also termed “fear conditioning”), as a special case of associative learning, is a long-standing model of anxiety disorders; accordingly, threat extinction learning underlies treatments such as exposure therapy [4]. Consequently, the phenomenon of associative learning—whether in the form of classical (Pavlovian) or operant (instrumental) conditioning—has been a subject of extensive empirical research. These empirical efforts have also been accompanied by a long tradition of computational modeling: since the seminal Rescorla-Wagner model [5], many other computational accounts of associative learning have been put forward [6–10], together with software simulators that implement these theories [11, 12]. As this space of models grows larger and more sophisticated, the aforementioned challenge of designing informative experiments becomes apparent. Here, we investigate how this challenge can be met through theory-driven, computational methods for optimizing associative learning experiments.

Similar methods of optimal experimental design have been developed in various subfields of biology and cognitive science concerned with formal modeling; for example, in systems biology [13, 14], neurophysiology [15, 16], neuroimaging [17, 18], psychology [19, 20], and behavioral economics [21, 22]. These efforts have generally demonstrated the potential benefits of computational optimization in experimental design. Yet, in research on associative learning, there is a lack of tools for design optimization, although the foundation for this development has been laid through extensive computational modeling. We therefore explore the potential of computationally optimizing experimental designs in associative learning studies, as opposed to the common practice of manually designing such experiments.

In optimizing experimental designs we usually need to rely on prior information: formalizing this notion is the essence of the Bayesian experimental design framework [23]. Bayesian experimental design is the basis of the aforementioned methods in other domains, and it also underlies our approach. This framework formalizes the problem of experimental design as maximizing the expected utility of an experiment. With this formulation we can integrate prior knowledge with explicit study goals to arrive at optimal designs. Moreover, upon observing new experimental data, the Bayesian design framework prescribes a principled manner in which to update the optimal design. However, Bayesian experimental design requires calculations of expected utility that usually result in analytically intractable integrals. To address this issue, we adopt a simulation-based approach to utility calculations [24, 25]. Using the simulation-based approach, we can flexibly choose the experiment structure, space of candidate models, goals of the study, and the planned analysis procedure, without the need to specify an analytically tractable utility function.

To illustrate the proposed design method, we apply it to the optimization of classical conditioning experiments, as a special case of associative learning. We first enable computational optimization by formalizing the structure of classical conditioning experiments and by identifying tunable design variables. Next, we propose low-dimensional design parameterizations that make optimization computationally tractable. We then generate optimized designs for three different types of study goals: namely, (1) the goal of accurately estimating parameters of a model, (2) the goal of comparing models with different learning mechanisms, and (3) the goal of comparing models with different mappings between latent states and emitted responses. Additionally, we perform the optimizations under varying levels of prior knowledge. Finally, we compare the optimized designs with commonly used manual designs drawn from the literature. This comparison reveals that optimized designs substantially outperform manual designs, highlighting the potential of the method.

## Methods

We can decompose the problem of designing experiments into three components: formalizing, evaluating, and optimizing. We will first outline each of these components of experimental design and introduce the main concepts. The details and the application to designing associative learning experiments are covered by the later sub-sections. Here we provide a non-technical description of the method, with the more formal treatment available in the S1 Appendix.

**Formalizing experiments**. involves specifying the *experiment structure* and parameterizing it in terms of *design variables*. Design variables are the tunable parameters of the experiment (e.g., probabilities of stimuli occurring), and the experiment structure is a deterministic or stochastic mapping from the design variables to *experiment realizations* (e.g., sequences of stimuli). The relationship between experiment structure, design variables, and experiment realizations, is analogous to the relationship between statistical models, their parameters, and observed data, respectively. Importantly, much like statistical models, experiment structures can be parameterized in various ways, some more amenable to design optimization than others.

**Fig 1:**
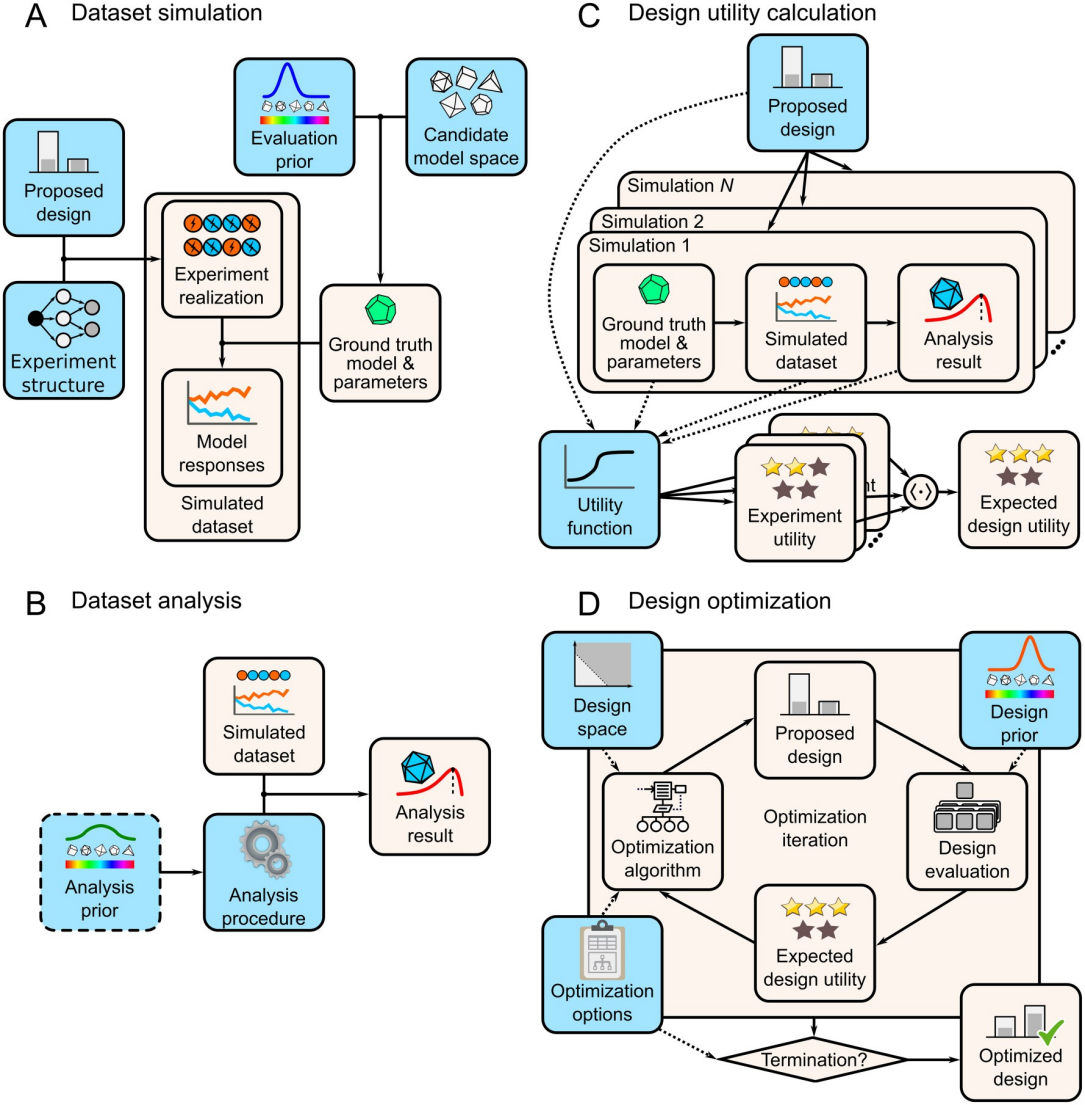
The simulation-based method of evaluating and optimizing experiment designs. Note: light-blue elements denote required user inputs. (A) Dataset simulation process generates experiment realizations (e.g., cue-outcome sequences) and model responses (e.g., predicted physiological or behavioral responses), which together form a simulated dataset. (B) Dataset analysis applies the user-specified analysis procedure to the simulated dataset and produces analysis results (e.g., model evidence or parameter estimates). If the analysis procedure is Bayesian, an additional analysis prior needs to be specified. (C) The calculation of expected design utility requires simulating and analyzing a number of datasets. The user-defined utility function—which can depend directly on the design or on other simulation-specific quantities—provides the utility value for each simulated experiment. The expected design utility is obtained by averaging experiment-wise utilities. (D) Design optimization proceeds in iterations: the optimization algorithm proposes a design from the design space, the design is evaluated under the design prior, and the expected design utility is passed back to the optimizer. If the optimization satisfies the termination criterion (which is one of the user-defined optimization options), the optimizer returns the optimized design.

**Evaluating experiments**. consists of calculating the expected utility for evaluated *designs*. Calculation of expected utility requires us to integrate across the distribution of datasets observable under our assumptions; however, this calculation generally results in analytically intractable integrals. To tackle this issue, we adopted the simulation-based approach to the evaluation of expected utility [24, 25]. The simulation-based approach entails three steps: simulating datasets, analyzing datasets, and calculating the expected utility (Fig 1A–1C).

Simulated datasets consist both of experiment realizations and *model responses* (Fig 1A). Experiment realizations are sampled from the experiment structure using the design being evaluated. In order to sample model responses, we first need to specify a *candidate model space* and an *evaluation prior*. A candidate model space is a set of generative statistical models, which encode hypotheses about possible data-generating processes underlying the phenomenon under study. An evaluation prior is a probability distribution that encodes the *a priori* plausibility of candidate models and their parameters. Using the evaluation prior, we can sample a candidate model and its parameters, which together comprise the *ground truth*. Using the ground truth model and the experiment realization, we can now sample model responses, thus completing the simulated dataset.

With the simulated dataset we can perform the planned *analysis procedure* (Fig 1B). This is the procedure that we plan to conduct with the dataset obtained in the actual experiment. Any type of statistical analysis can be used here, but Bayesian analyses seem particularly appropriate, since specifying generative models and priors is already a prerequisite of the design evaluation procedure. If we employ a Bayesian analysis, an additional prior needs to be specified—the *analysis prior*. Unlike the evaluation prior—which should reflect the experimenter’s best guess about the state of the world, and can be as informative as deemed appropriate—the analysis prior should be acceptable to a wider audience, and therefore should not be overly informative or biased [26]. The analysis procedure—Bayesian or otherwise—will yield an *analysis result*; for example, a parameter estimate (in estimation procedures) or a discrete choice of the best-supported model (in model selection procedures).

Having performed the analysis on the simulated dataset, we can now calculate the *experiment utility* (Fig 1C). The experiment utility is provided by the *utility function*, which encodes the goals of the study, and maps the values of design variables to utility values. Although we are interested in how the utility depends on the design, the utility function may generally depend not only on the direct properties of the design (e.g., experiment duration and cost) but also on quantities influenced by the evaluation prior or the analysis procedure, e.g., the analysis result and the ground truth. For example, when the goal is accurate parameter estimation, the utility function can be based on the discrepancy between the true parameter value (ground truth), and the obtained parameter estimate (analysis result), neither of which are direct properties of the design. Finally, to obtain the *expected design utility*, we simulate a number of experiments using the same design, calculate the experiment utility for each experiment, and average the utility values across experiments.

**Optimizing experiments**. requires finding the values of design variables that maximize the expected utility under our evaluation prior (please note, we will refer to the evaluation prior used in design optimization as the *design prior*, to avoid confusion when a design is optimized using one prior, but ultimately evaluated using a different prior). The principle of maximizing expected design utility is the core idea of Bayesian experimental design, which is simply the application of Bayesian decision theory to the problem of designing experiments [23, 27, 28]. Practically, maximizing expected design utility can be achieved using optimization algorithms (Fig 1D). We first specify the domain over which to search for the optimal design—the *design space*. The design space is defined by providing the range of feasible values for the design variables, which must also take into account any possible constraints on these variables. Next, we specify the optimization objective function, which is in this case the expected design utility, evaluated through the previously described simulation-based approach. Lastly, we specify the parameters of the optimization algorithm, e.g., the termination criterion. The optimization proceeds in iterations that consist of the algorithm proposing a design, and obtaining back the expected design utility. After each iteration, the termination criterion is checked: if the criterion is not fulfilled, the algorithm continues, by proposing a new design (based on the previously observed utilities). Ultimately, the algorithm returns an *optimized design*, which—importantly—might not be the *optimal design*, depending on the success of the search.

### Formalizing associative learning experiments

As outlined, in order to optimize associative learning experiments, we first need to formalize their structure. Here we consider the paradigm of classical conditioning, as a special case of associative learning, where associations are formed between presented cues and outcomes. There are many design variables that need to be decided in implementing a conditioning experiment, such as physical properties of the stimuli, or presentation timings. However, learning is saliently driven by statistical contingencies between cues and outcomes in the experiment; consequently, most computational models of associative learning capture the effect of these contingencies on the formation of associations. Therefore, we focus on the statistical properties of the experiment as the design variables of interest.

**Fig 2:**
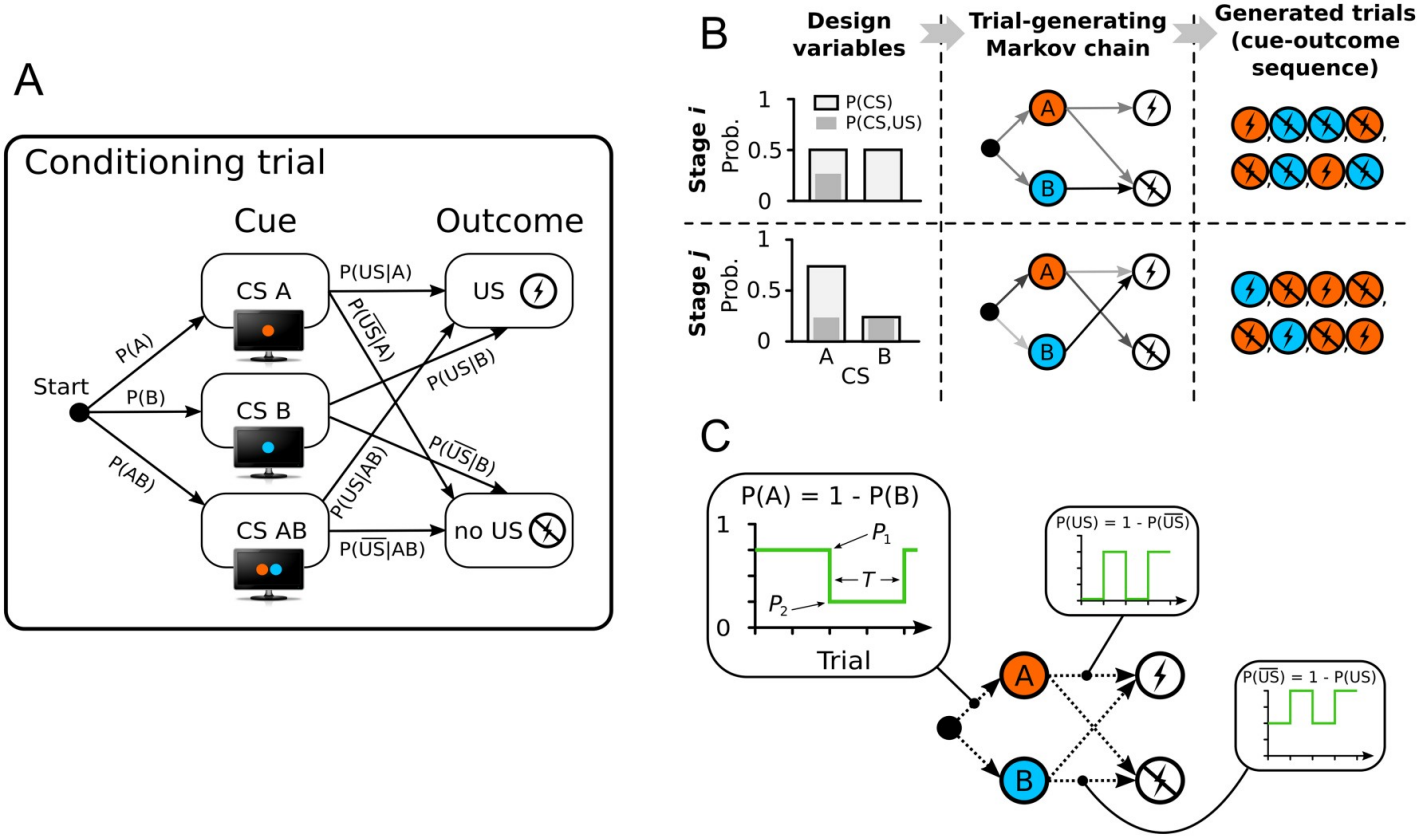
Formalizing and parameterizing the structure of classical conditioning experiments. (A) A conditioning trial can be represented as a Markov chain, parameterized by the probabilities of presenting different cues (e.g., colored shapes), and transition probabilities from cues to outcomes (e.g., delivery of shock vs. shock omission). (B) Stage-wise parameterization of conditioning experiments. Trials in each stage are generated by a Markov chain with constant transition probabilities. The transition probabilities of each stage are tunable design variables. (C) Periodic parameterization. The transition probabilities in the Markov chain are determined by square-periodic functions of time (i.e., trial number). The design variables are the two levels of the periodic function and its half-period.

We formalize a classical conditioning experiment as a sequence of conditioning trials. A conditioning trial consists of presenting one of the cues (i.e., conditioned stimulus, CS), followed—with some probability—by an aversive or appetitive outcome (i.e., unconditioned stimulus, US). If each trial’s cue-outcome pair is sampled independently, and if the probability of the outcome depends only on the cue, then each trial can be represented as a Markov chain (Fig 2A). In this formalization, the cue probabilities *P*(CS) and conditional outcome probabilities *P*(US|CS) are the transition probabilities of the trial-generating Markov chain. These transition probabilities represent the design variables to be optimized.

Allowing the transition probabilities of each trial to be independently optimized results in a high-dimensional optimization problem, even with a modest number of trials. However, the majority of existing experimental designs for studying associative learning vary the cue-outcome contingencies only between experiment stages (i.e., contiguous blocks of trials), and not between individual trials. Examples of such designs include forward (Kamin) blocking [29], backward blocking [30], reversal learning [31], overexpectation [32], overshadowing [33], conditioned inhibition [33], and latent inhibition [34]. Constraining the designs to allow only for contingency changes between stages can dramatically reduce the number of design variables, making optimization tractable. Therefore, the first design parameterization of conditioning experiments that we introduce here is the *stage-wise parameterization*. In this parameterization the experiment is divided into stages, with each stage featuring a trial-generating Markov chain with constant transition probabilities (Fig 2B). The number of trials per stage is assumed to be known, and the tunable design variables are stage-wise cue probabilities *P*(CS) and conditional outcome probabilities *P*(US|CS) (some of these design variables can further be eliminated based on constraints). The aforementioned motivating designs from the literature are special cases of the stage-wise parameterization, but this parameterization also offers enough flexibility to go beyond classical designs.

However, if we are interested in associative learning in environments with frequent contingency changes, the stage-wise parameterization may require many stages to implement such an environment, and would thus still lead to a high-dimensional optimization problem. To address this scenario, we introduce the *periodic parameterization*, which is a parameterization based on periodically-varying contingencies (Fig 2C). In this parameterization, each cue probability *P*(CS) and conditional outcome probability *P*(US|CS) is determined by a periodic function of time (i.e., trial number). Specifically, we use a square-periodic function parameterized with the two levels *P*_1_ and *P*_2_, between which the probability cycles, and the length of the half-period *T*. This parameterization allows expressing designs with many contingency changes using a small number of design variables, when compared with an equivalent stage-wise design; however, unlike the stage-wise parameterization, the periodic parameterization constrains contingency changes to occur in regular intervals and only between two fixed values.

### Evaluating associative learning experiments

Having formalized the structure of classical conditioning experiments, we now specify the procedure to evaluate their expected utility using the simulation-based approach. To simulate a classical conditioning dataset, we first sample an experiment realization—i.e., a sequence of cues (CSs) and corresponding outcomes (USs)—using the experiment structure and the evaluated design. We then sample the ground truth model of associative learning and its parameter values from the evaluation prior. Using the sampled CS-US pairs, sampled parameter values, and the generative model, we generate the conditioned responses (CRs). These trial-wise triplets of CSs, USs, and CRs constitute a single simulated dataset.

With this simulated dataset we can proceed to perform the analysis that we are planning to do with the real dataset that will be collected. In scenarios presented here, we simulate and analyze only single-subject data. We do so for two reasons. First, the single-subject scenario provides more difficult conditions for statistical inference, due to the limited amount of data. Second, the single-subject analysis is especially relevant from a translational view, which considers clinical inferences that need to be done for each patient individually.

For the analysis, we can specify any procedure that is afforded by the simulated dataset, but computationally demanding analyses can lead to long design evaluation running times. In the current article, we focus on two model-based types of analyses that are common in the modeling literature on associative learning: parameter estimation within a single model, and model selection within a space of multiple models. For parameter estimation, we use the maximum a posteriori (MAP) approach with uniform priors (which is equivalent to maximum likelihood estimation (MLE)), and for model selection we use the Bayesian information criterion (BIC), which takes into account both the model fit and model complexity. Both procedures are computationally relatively inexpensive and were implemented using the existing ‘mfit’ toolbox for MATLAB [35]. Fully Bayesian inference with proper analysis priors could also be used here, as long as the inference procedure is not overly computationally expensive.

Finally, with the results of the analysis on the simulated dataset, we can assess the utility of the experiment. The utility function should be determined by the goals of the study. In this article, for parameter estimation analyses, we aim to minimize the absolute error between the parameter estimate and the ground truth value, and for model selection analyses, we aim to minimize the misidentification of the ground truth model (i.e., model selection error or 0-1 loss).

### Optimizing associative learning experiments

So far, we have formalized the structure of associative learning experiments, and provided a method of assessing their utility by simulation. We are now in a position to specify a method of optimizing the design of such experiments.

Although the simulation-based approach to design evaluation is instrumental in providing flexibility to the user, it can yield difficult optimization problems. Evaluating the expected design utility using stochastic simulations gives noisy estimates of utility and can often be computationally expensive. Furthermore, we generally cannot make strong assumptions about the utility function (e.g., convexity) and we usually do not have access to the derivatives of the function. A state-of-the-art optimization algorithm for such scenarios is Bayesian optimization [36]. Bayesian optimization has already been effectively used in experimental design problems [22, 37], and it was therefore our optimizer of choice. Bayesian optimization is a global optimization algorithm which uses a surrogate probabilistic model of the utility function to decide which values of optimized variables to evaluate next; this allows it to efficiently optimize over expensive utility functions, at the cost of continuously updating the surrogate model (see S1 Appendix for technical details). In results presented here, we used the MATLAB bayesopt function (from the ‘Statistics and Machine Learning Toolbox’) which implements Bayesian optimization using a Gaussian process as the surrogate model.

To fully specify the design optimization problems, we also need to define the design space, the design prior, and the optimization options. The design space over which the optimization was performed depended on the design parameterization. For the stage-wise parameterization, each transition probability could range from 0 to 1, with the constraint that the sum of transition probabilities from a given starting state is 1. For the periodic parameterization, the two probability levels *P*_1_ and *P*_2_, and the half-period *T* (expressed as a fraction of the total trial number) also ranged from 0 to 1. For the periodic parameterization, the design is constrained such that all the transition probabilities from a given state have the same half-period, and all *P*_1_ and *P*_2_ probabilities have to sum up to 1, respectively. The design priors were determined in a problem-specific manner, and are described together with the application scenarios in the Results section. In general, the design prior should be based on the information available from the relevant literature or from existing data. Lastly, the optimization options depend on the specifics of the chosen optimization algorithm (for Bayesian optimization, details are provided in the S1 Appendix). A common optimization option is the termination criterion: we used a fixed maximum number of optimization iterations as the criterion, but the maximum elapsed time, or the number of iterations without substantial improvement can also be used.

### Workflow for evaluating and optimizing associative learning experiments

Finally, we propose a workflow for employing the described simulation-based method in the evaluation and optimization of associative learning experiments. The proposed workflow is as follows (the required user inputs are italicized; see also Fig 1):

1. **Specify the assumptions and goals of the design problem**. In this step we need to specify the components of the design problem which are independent of the specific designs that will be evaluated. In particular, we specify our *candidate model space*, *evaluation priors* over the models and their parameters, *analysis procedure*, *analysis priors*, and the *utility function* we wish to maximize.
2. **Evaluate pre-existing reference designs**. If there are pre-existing *reference designs*, it is advisable to first evaluate their expected utility. If these designs already satisfy our requirements, we do not need to proceed with design optimization. In this step we first formalize reference designs, and then we use the simulation-based approach to evaluate their expected utility under the assumptions made when specifying the design problem (step 1).
3. **Optimize the design**. If existing reference designs do not satisfy our requirements, we proceed with obtaining an optimized design. In this step we first specify the *experiment structure* and its *design variables* (both tunable and fixed), the *design space*, the *design priors*, and *optimization options*. Having specified these, we run the design optimization until the termination criterion is fulfilled, and then inspect the resulting design.
4. **Evaluate the optimized design**. We take the optimized values of design variables obtained in step 3, and use them to specify the optimized design. Then we obtain the expected utility of the optimized design in the same simulation-based manner as we did for the reference designs (in step 2), and inspect the obtained results.
5. **Compare the reference and optimized designs**. Having evaluated the reference and optimized designs through simulations (in steps 2 and 4, respectively), we can now compare their expected utilities. For example, if we are aiming for accurate parameter estimates, we can inspect the design-wise distributions of estimation errors, and if we are aiming for accurate model selection, we can inspect the design-wise confusion matrices (and corresponding model recovery rates). If the optimized design does not satisfy our requirements, we can go back to step 3; for example, we can modify the experiment space or run the optimization for longer. If the optimized design satisfies our requirements, we can proceed to its implementation.

For one of the simulated scenarios presented in the Results section (Scenario 2), we illustrate the described workflow with an executable MATLAB notebook provided on Zenodo at https://doi.org/10.5281/zenodo.3582947.

## Results

We validated the proposed method in three scenarios, with one scenario targeting the goal of accurate parameter estimation, and the other two targeting the goal of accurate model selection. The overview of user inputs employed in these scenarios is given in Table 1. In each scenario we compared optimized designs with a reference design drawn from prior work in associative learning. In order to evaluate the design’s accuracy of parameter estimation or model selection, it is necessary to know the ground truth, i.e., to sample a data-generating model and its parameter values from the evaluation prior. Therefore, we have evaluated the designs through simulations, as is common in the field of experimental design. To illustrate the benefit of optimized designs, we report estimates of the difference in accuracy between the designs, and the associated 95% confidence intervals. We do not quantify the benefit of optimized designs using classical *p*-values because the number of simulations (sample size) can be made arbitrarily large, such that optimized designs can be made “significantly” better than reference designs even if the differences are trivially small [38].

**Table 1:** Overview of user inputs for the three simulated scenarios.

User inputs	Scenario 1	Scenario 2	Scenario 3
**Candidate model space**	RW	RW, KRW	RW(*V*), RWPH(*V*), RWPH(*α*), RWPH(*V* + *α*)
**Analysis procedure**	Maximum likelihood parameter estimation	Model selection using BIC	Model selection using BIC
**Utility function**	Absolute error of learning rate (*α*) estimate	Model selection accuracy	Model selection accuracy
**Analysis prior**	Uniform over *α*	Uniform over models and parameters	Uniform over models and parameters
**Reference design**	Acquisition followed by extinction of equal length	Backward blocking	Reversal learning
**Evaluation prior**	Point priors on low, middle and high *α* (LA, MA, HA)	Uniform over models with point priors on parameters (from [39])	Uniform over models with point priors on parameters (from [31])
**Optimized experiment structure**	One cue with periodically varying contingency	Two stages with three cues (A, B, AB) and stage-wise contingencies	Two stages with two cues (A, B) and stage-wise contingencies
**Design space**	Two contingencies (*P*_1_, *P*_2_) and the period (*T*) of their switching (3 variables)	For each stage *s* and cue *X*: *P*_*s*_(*X*), *P*_*s*_(US|*X*) (10 non-redundant variables)	For each stage *s* and cue *X*: *P*_*s*_(*X*), *P*_*s*_(US|*X*) (6 non-redundant variables)
**Design priors**	Either a point prior over *α* coinciding with evaluation prior (PA) or a vague prior (VA)	Uniform over models with either a point (PP) or vague (VP) prior over parameters	Uniform over models with either a point (PP) or vague (VP) prior over parameters

The role of user inputs is clarified by Fig 1. See the Methods section for details. Analysis priors are used in fitting models, design priors are used to simulate data when optimizing designs, and evaluation priors are used to simulate data when evaluating both reference and optimized designs.

Furthermore, we wanted to investigate the effect of different amounts of background knowledge, i.e., to perform a sensitivity analysis with respect to the design prior, while holding the ground truth (evaluation prior) constant. Therefore, we optimized designs both under a vague (weakly-informative) design prior and under a strongly-informative design prior. The designs were then evaluated using point evaluation priors, representing an assumed ground truth. With these two extreme design priors, we obtain, respectively, approximate lower and upper bounds on the expected design utility of optimized experiments. However, please note that while in a methodological study—like this one—it can be advantageous to disambiguate between the design prior and the evaluation prior, in practical applications these two priors should coincide, with both of them expressing the experimenter’s knowledge base at the time of designing the experiment.

### Optimizing for accuracy in parameter estimation

An early, but still widely influential model of classical conditioning is the Rescorla-Wagner (RW) model [5]. A parameter of particular interest in this model is the learning rate *α* [40]. For example, the learning rate of the RW model fitted to human learning data in volatile conditions has been found to correlate with trait anxiety [41]. Therefore, in Scenario 1, we optimized designs with the goal of accurately estimating the RW learning rate *α*.

As ground truth, three different point evaluation priors on learning rate were used: low alpha (LA, *α* = 0.1), middle alpha (MA, *α* = 0.2), and high alpha (HA, *α* = 0.3). Three types of experimental designs were compared under these three evaluation priors using the absolute estimation error metric. All three designs have the same structure, which is simple but sufficient to estimate the learning rate: the probability of the CS being followed by a US (*P*(US|CS)) is a periodic function of time, and it switches between two discrete values. Therefore, there are three variables that specify the design: the two reinforcement probabilities, and the period with which these two probabilities are switched.

The first evaluated design is the (manual) reference design (denoted REF), which was taken from existing literature rather than being computationally optimized. Here we chose the often-used classical conditioning design in which an association is first acquired under partial reinforcement of a CS with a US (acquisition stage), and then extinguished by ceasing the reinforcement of the CS (extinction stage) [42]. The probabilities of reinforcement were 0.5 and 0 in the acquisition and the extinction stages, respectively. The switch between the stages occurred only once, at the middle of the experiment. The second design is the design optimized under a vague prior over the learning rate *α* (denoted VA-OPT). The vague prior here is implemented as a uniform prior over *α*. This prior reflects the situation when there is little information on the parameter of interest at the planning stage of the experiment. Under this prior we are estimating a lower bound on the accuracy of optimized experiments. The third design is the design optimized under the exact point prior on the learning rate *α* (denoted PA-OPT). This prior reflects the situation where we have perfect knowledge of the ground truth learning rate, and it therefore results in different specific designs for the LA, MA, and HA evaluation priors. Although in practice the ground truth is not known, under this prior we can estimate an upper bound on the accuracy of optimized experiments.

The optimized designs were obtained by running Bayesian optimization for 300 iterations, with the absolute estimation error of the learning rate being minimized. In each iteration, the estimation error was evaluated using 32 datasets simulated from the RW model, with parameters being randomly drawn from the design prior. Using 32 CPU cores at 2.5 GHz, average time to obtain the optimized design was 0.5 h, with the average being taken across optimizations with different design priors. The reference design and the optimized designs were evaluated in additional 256 simulations per each combination of the evaluation prior (ground truth) and design.

**Fig 3:**
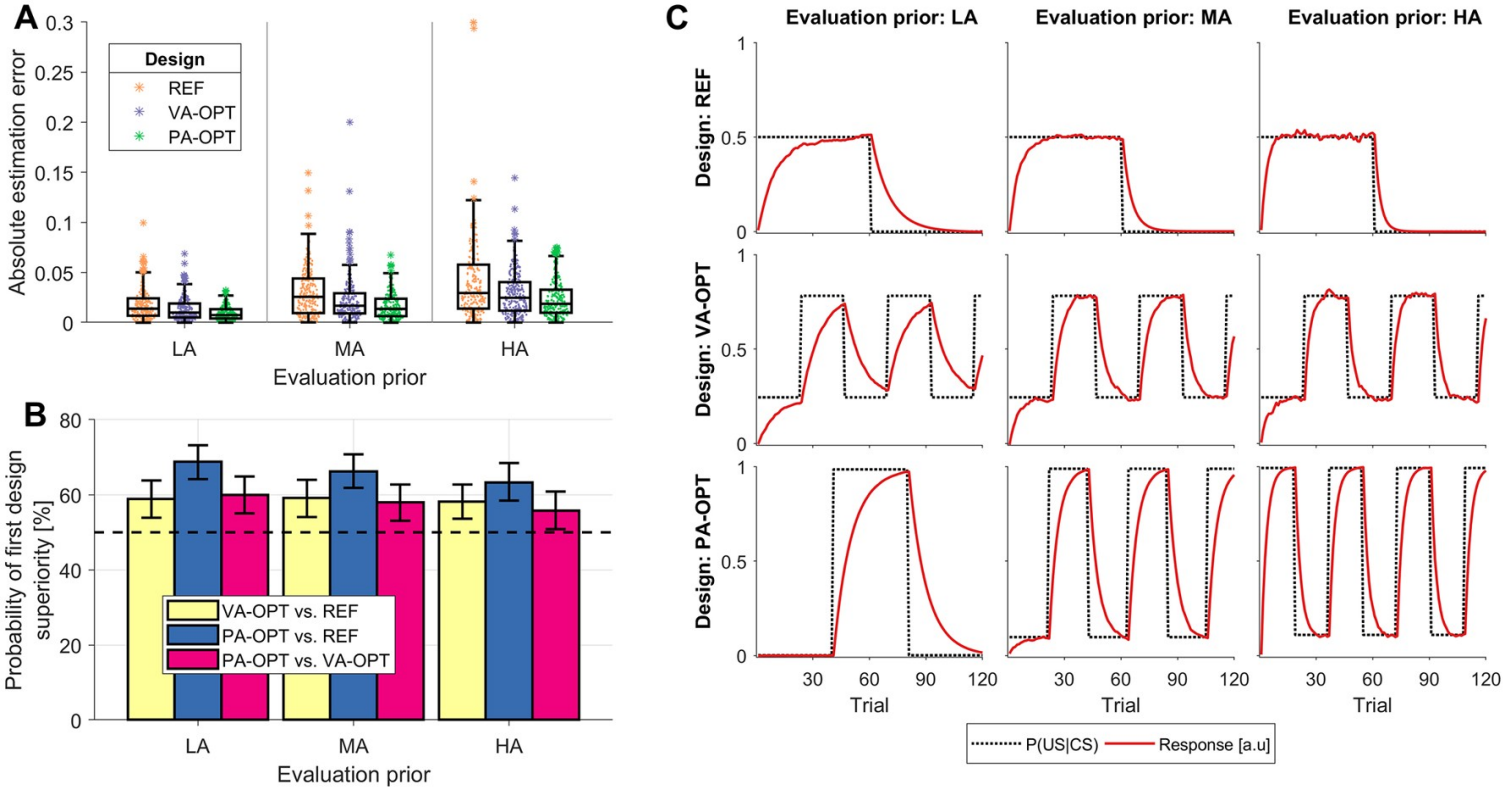
Design evaluation in Scenario 1: RW model learning rate estimation. Three designs—reference acquisition-extinction design (REF), design optimized under a vague prior (VA-OPT), and design optimized under a point prior (PA-OPT)—are evaluated under the low (LA), middle (MA), and high (HA) value of the learning rate alpha. (A) Distribution of absolute errors in estimating the learning rate. (B) Pair-wise comparison of design accuracy expressed as the probability of the first design in the pair being superior (i.e., having lower estimation error). Error bars indicate bootstrapped 95% CI and 50% guideline indicates designs of equal quality. (C) Comparison of model responses (red full line) to the contingencies (black dashed line) obtained under different designs and different evaluation priors.

The results of the design evaluation are shown in Fig 3. We first inspect the distribution of absolute estimation errors under different designs and evaluation priors (Fig 3A). Under all designs, the estimation errors were relatively small (with almost all simulations yielding errors below 0.1). This can be attributed to sufficient number of simulated trials and moderate levels of observation noise. Nevertheless, even in such favorable conditions, the REF design yielded the largest estimation errors, followed by the VA-OPT design, and the PA-OPT design. This suggests that including even weak prior knowledge (as in the VA-OPT design) can improve parameter estimation accuracy, with more informative prior knowledge providing further improvements (as in PA-OPT).

Next, we quantified the relative differences in design utility, by estimating the probability that a randomly sampled experiment from one design will yield higher utility than an experiment sampled from another design (i.e., the probability of superiority). These probabilities were estimated for all pairs of designs by computing the non-parametric common language effect sizes (CLES) [43], which indicate designs of equal expected utility with CLES = 50%. The 95% CIs for CLES estimates were obtained using the percentile bootstrap method [44], with 1000 bootstrap samples. The appropriateness of the percentile bootstrap method was verified by inspecting bootstrap distribution histograms, which did not exhibit substantial bias or skew. The design evaluation results are shown in Fig 3B and they confirm that the VA-OPT design is more probable to yield lower estimation errors than the REF design, under all evaluation priors: the probabilities of VA-OPT being superior are 58.9% (95% CI: [53.9%–63.8%]), 59.2% (95% CI: [54.1%–64.0%]), and 58.2% (95% CI: [53.6%–62.7%]) for the LA, MA, and HA evaluation priors, respectively. Moreover, the the PA-OPT design is probable to be superior–under all evaluation priors–both to the REF design (LA—68.8%, 95% CI: [64.2%–73.2%]; MA—66.3%, 95% CI: [61.8%–70.8%]; HA—63.3%, 95% CI: [58.4%–68.5%]) and to the VA-OPT design (LA—60.0%, 95% CI: [55.0%–64.9%]; MA—58.0%, 95% CI: [53.1%–62.7%]; HA—55.8%, 95% CI: [50.8%–60.8%]).

Finally, we inspect the obtained designs and the corresponding model responses in Fig 3C. The REF design and the VA-OPT design are the same under all evaluation priors, since they do not take into account the ground truth learning rate, whereas the PA-OPT designs are tuned to the exact parameter value. Compared to the REF design, the VA-OPT design uses more frequent contingency switches, allowing for more accurate estimation of the learning rate under a wide range of possible values. The PA-OPT design adapts the frequency of contingency switches to the learning rate, with low learning rates being better estimated from longer periods of stability, and higher learning rates being better estimated from frequent transitions.

### Optimizing for accuracy in model selection

The basic RW model explains many aspects of associative learning, but it fails to account for many others [45]. For this reason, various extensions of the basic RW model have been proposed in the literature. One example is the Kalman Rescorla-Wagner (KRW) model [7, 9, 39], which is a probabilistic extension of the RW model. Another example is the hybrid Rescorla-Wagner-Pearce-Hall (RWPH) model [31], which maintains separate dynamic learning rates (termed “associabilities”) for all the cues. The growing space of theoretical models of associative learning highlights the need for experiments that can efficiently expose the differences between these accounts. Therefore, in Scenarios 2 and 3, we optimized designs to achieve the goal of accurate model selection (i.e., how often the selected winning model coincides with the ground truth model). The model space of Scenario 2 is comprised of models with different learning mechanisms, whereas the model space of Scenario 3 entails not only models with different learning mechanisms, but also models with different mappings from latent model states to conditioned responses.

Scenario 2 is based on the simulation study of Kruschke (2008) [39], which compares the RW and the KRW model using the backward blocking design. The backward blocking design is comprised of two stages. In the trials of the first stage, only the reinforced compound cue AB is presented. In the trials of the second stage, only one of the elements of the compound (e.g., A) is presented and reinforced. In the test stage, the single element cues—A and B—are tested separately in non-reinforced trials. This design has been used to compare the RW and KRW models because they make different predictions about test responding to the cue that was not presented in the second stage (here, cue B). According to the RW model, responding to cue B should be unchanged, as this model allows cue-specific associations to change only when the cues are presented. In contrast, the KRW model predicts that the responding to cue B at test will be diminished (blocked), because the model has assigned the credit for reinforcements to the cue A (i.e., during the first stage a negative correlation between the associative weights for element cues is learned). Due to the opposing model predictions for this design, and its previous use in literature, we chose it as the reference (REF) design in Scenario 2.

Scenario 3 is based on the empirical study of Li et al. (2011) [31], which compared the RW model (labeled RW(*V*)) and three variants of the RWPH model. The variants of the RWPH model were formulated to emit different model quantities as the conditioned response: associative weights (RWPH(*V*)), associabilities (RWPH(*α*)), or a mixture of weights and associabilities (RWPH(*V* + *α*)). In order to distinguish between these models Li et al. (2011) [31] employed a reversal learning design, and this is the design we used as the reference (REF) design in Scenario 3. The design is comprised of two stages: in the first stage one cue is partially reinforced, while the other is not, and in the second stage the two cues switch their roles. This design is intuitively appealing for the goal of discriminating between the RW and RWPH models: at the point of contingency reversal, the learning rate in the RWPH model increases due to large prediction errors, while in the RW model the learning rate is constant.

In both Scenario 2 and 3 the structure and parameterization of the optimized designs was the same: the experiment had two stages (with equal number of trials), and the cue-outcome contingencies were kept constant within each stage. Hence, the stage-wise design variables were the CS presentation probabilities and the probabilities of the US conditional on the CS. To keep a close comparison with the reference designs, Scenario 2 included two CSs (A and B) and their compound (AB), whereas Scenario 3 only included two elemental CSs. Similarly to Scenario 1, we obtained optimized designs under two types of design priors: a vague prior on model parameters (in the VP-OPT design) and an exact point design prior on model parameters (in the PP-OPT design). The vague priors were either uniform (for bounded parameters) or only weakly informative (see S1 Appendix for details). In Scenario 2 the point priors were set at the same values as in the simulations of Kruschke (2008) [39]. The point priors in Scenario 3 were set to the best fitting parameter values of the RWPH(*V* + *α*) model reported in the study of Li et al. (2011) [31] (the best fitting parameters of other models are not reported in their study); for other models in this scenario, we were able to use subsets of the RWPH(*V* + *α*) parameters, since the models were nested. Importantly, even when the exact point priors on model parameters were used in the design optimization (PP-OPT designs), the simulated datasets were sampled in equal proportions from all candidate models (implying a uniform prior over models).

**Fig 4:**
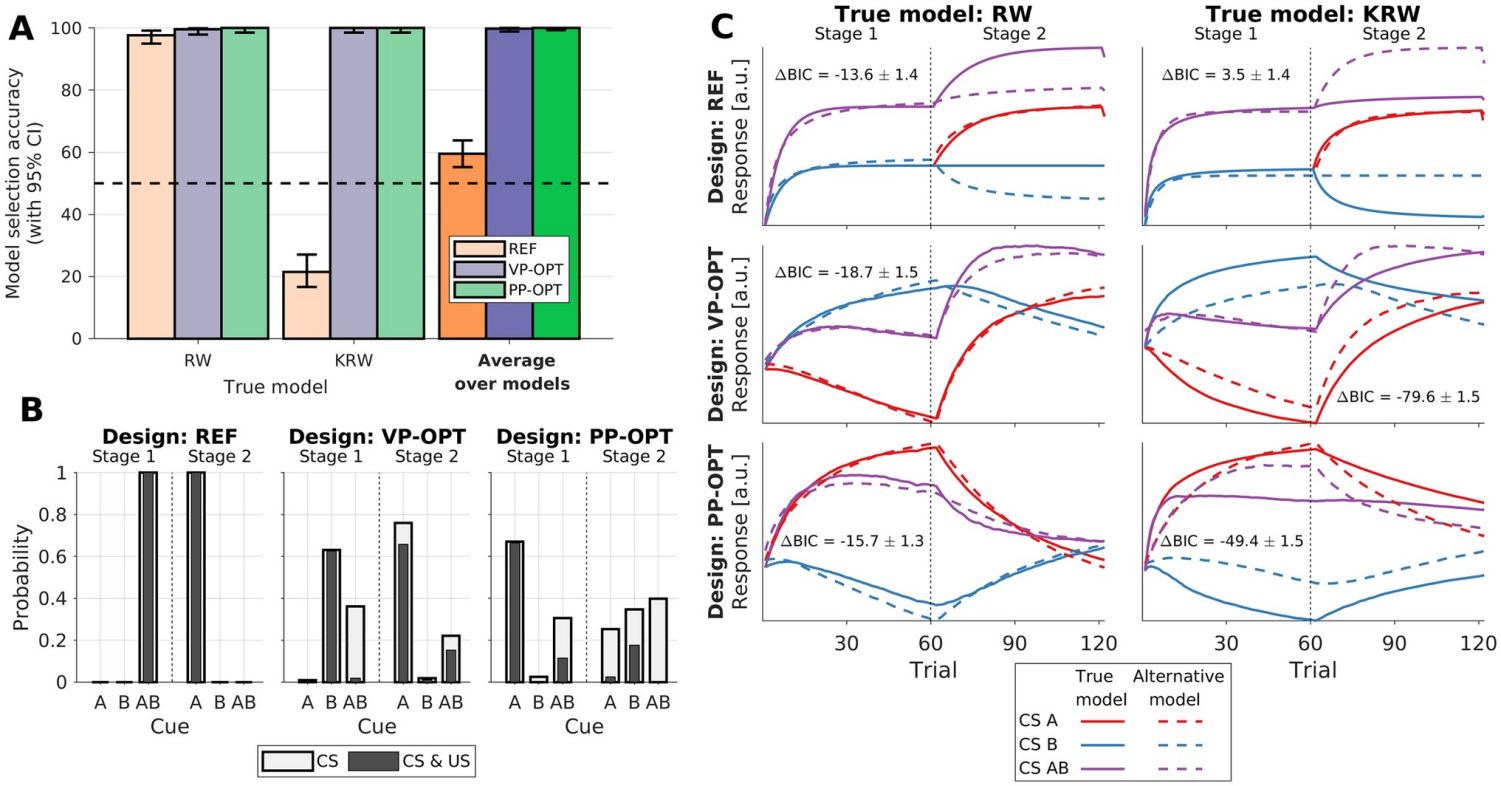
Design evaluation in Scenario 2: Selection between RW and KRW models. Three designs—reference backward blocking design (REF), design optimized under a vague prior (VP-OPT), and design optimized under a point prior (PP-OPT)—are evaluated under the ground truth model being either the RW or the KRW model. (A) Model selection accuracy (mean and the Clopper-Pearson binomial 95% CI). Horizontal guideline indicates chance level. Darker bars summarize results across ground truth models. (B) Values of the design variables in the two stages of the experiment: cue probabilities *P*(CS) and joint cue-outcome probabilities *P*(CS, US). (C) Comparison of fitted model responses obtained under different designs (rows) and different ground truth models (columns). Inset labels give the average difference in BIC (±SEM) between the fit of the true model and the alternative model (more negative values indicate stronger evidence in favor of the true model).

**Fig 5:**
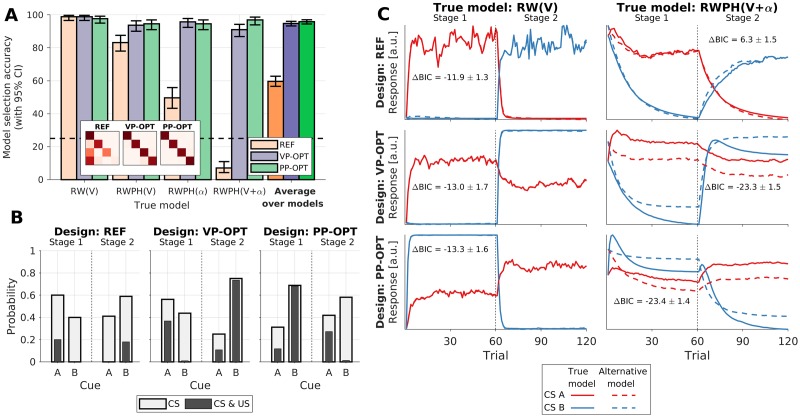
Design evaluation in Scenario 3: Selection between RW and RWPH models. Three designs—reference reversal learning design (REF), design optimized under a vague prior (VP-OPT), and design optimized under a point prior (PP-OPT)—are evaluated under the ground truth model being either the RW(*V*), RWPH(*V*), RWPH(*α*), or RWPH(*V* + *α*). (A) Model selection accuracy (mean and the Clopper-Pearson binomial 95% CI). Horizontal guideline indicates chance level. Darker bars summarize results across ground truth models. Inset shows the confusion matrix between the ground truth model (rows) and the selected model (columns). (B) Values of the design variables in the two stages of the experiment: cue probabilities *P*(CS) and joint cue-outcome probabilities *P*(CS, US). (C) Comparison of fitted RW(*V*) and RWPH(*V* + *α*) model responses obtained under different designs (rows) and these two models as assumed ground truth (columns). Inset labels give the average difference in BIC (±SEM) between the fit of the true model and the alternative model (more negative values indicate stronger evidence in favor of the true model). Note: the model responses are nearly identical when the RW(*V*) model is true, because this model is a special case of the alternative RWPH(*V* + *α*) model.

In both Scenario 2 and 3, we ran the Bayesian design optimization for 300 iterations and in each iteration the utility function (model selection accuracy) was evaluated using 32 datasets simulated from each of the candidate models. Average optimization times (across different design priors) were 5.2 h in Scenario 2, and 14.1 h in Scenario 3. The optimized designs and the reference designs were evaluated in additional 256 simulated experiments per each combination of the ground truth model and design. Additionally, we computed the responses of models fitted to the evaluation simulations. For visualization, the responses of fitted models to each CS were computed in all the trials, even though in the simulated experiments only one of the CSs was presented in each trial. The evaluation results for Scenario 2 and 3 are shown in Figs 4 and 5, respectively.

For Scenario 2, model selection accuracies of reference and optimized designs are shown in Fig 4A. Although the REF design yields accuracies above chance level (mean: 59.6%, 95% CI: [55.2%–63.9%]), optimized designs provide substantial improvements. Using a vague prior, the VP-OPT design provides a lower bound on the accuracy expected with an optimized design; nonetheless, even this lower bound provides near perfect accuracy (mean: 99.8%, 95% CI: [98.9%–100.0%]). We can quantify the relative improvement using the odds ratio (OR) as the effect size (please note: the value OR = 1.0 indicates designs of equal expected utility, and the 95% CIs were obtained using the Woolf’s method with the Haldane-Anscombe correction [46]). The VP-OPT design greatly increases the odds of correctly identifying the ground truth model (OR: 346.8, 95% CI: [48.4, 2486.4]). Moreover, with the exact knowledge of model parameters, the PP-OPT design provides an upper bound on expected improvements. Due to the already near-perfect accuracy of the VP-OPT design, the PP-OPT design yielded similar accuracy (mean: 100.0%, 95% CI: [99.3%–100.0%]). The relative improvement of the PP-OPT design over the REF design is substantial (OR: 696.2, 95% CI: [43.2, 11208.0]). However, due to a ceiling effect, we do not observe a clear advantage of the PP-OPT design over the VP-OPT design (OR: 3.0, 95% CI: [0.1, 74.0]).

Similar results are observed when comparing the design accuracies in Scenario 3 (Fig 5A). Accuracy of the REF design is significantly above chance level (mean: 59.7%, 95% CI: [56.6%–62.7%]), but the designs optimized both under a vague and point prior provide greatly improved accuracies. The VP-OPT design yields both a high accuracy in absolute terms (mean: 94.8%, 95% CI: [93.3%–96.1%]) and a large improvement relative to the REF design (OR: 12.4, 95% CI: [9.1, 16.8]). The PP-OPT design also provides a high accuracy (mean: 95.9%, 95% CI: [94.5%–97.0%]), and an even larger improvement relative to the REF design (OR: 15.8, 95% CI: [11.3, 22.1]); however, the improvement relative to the VP-OPT design seems to be small (OR: 1.3, 95% CI: [0.8, 1.9]).

In both Scenario 2 and 3, we can observe that the largest gain in accuracy is realized when the true model is the most complex candidate model (KRW in Scenario 2, and RWPH(*V* + *α*) in Scenario 3). With the REF designs, data simulated from these more complex models is often attributed to simpler models (e.g., see confusion matrix in Fig 5A). Such misidentification occurs because both simple and complex models can account for the data from a simple design similarly well, but the model selection criterion (BIC in our case) penalizes complexity, thus biasing the selection towards simple models and even leading to accuracies below chance level, as seen in Figs 4A and 5A. This suggests that simple manual designs do not provide the conditions in which complex models could make predictions sufficiently distinct from simpler models. Hence, to achieve conditions in which candidate models make sufficiently diverging predictions, the simplicity of experimental designs may need to be sacrificed. Indeed, this is what we observe by comparing the values of design variables between the REF, VP-OPT, and PP-OPT designs, both in Scenario 2 (Fig 4B) and Scenario 3 (Fig 5B). In both cases, the optimized designs are arguably less parsimonious than the reference designs. However, the optimized designs also result in more distinct model responses, especially when the ground truth model is more complex.

For example, in Scenario 2, model responses of the fitted RW and KRW models (Fig 4C) are very similar for cues presented in the REF design (AB in stage 1, and A in stage 2), regardless of which model is the ground truth. We can observe a similar pattern in Scenario 3 (Fig 5C), where we show the responses of the RW(*V*) and RWPH(*V* + *α*) models, which are most often confused under the REF design. This is also reflected in the BIC differences between the true and the alternative models (ΔBIC), which are—under the REF design—in favor of the simple model regardless of the ground truth model. In contrast, model responses with optimized designs are more distinct when the ground truth model is complex, allowing for easier model discrimination in both scenarios. However, when the ground truth model is simple, model responses are similar even under optimized designs; yet, this is not a failure of the design optimization: when the predictions are similar, the simpler model will correctly be selected, since BIC favors parsimony. Similarly, it may seem surprising that the PP-OPT designs can yield evidence in favor of the true model that is weaker than in the VP-OPT designs (e.g., see Fig 4C) in terms of ΔBIC, even though the PP-OPT designs are based on stronger prior knowledge and provide similar or higher accuracies than VP-OPT designs. Again, this is not a failure of the optimization; instead, the procedure takes into account that our goal is maximizing the frequency of correct model identifications, rather than the subtly different goal of maximizing the expected evidence in favor of the true model. Altogether, these observations demonstrate that the optimization took into account not only the supplied model space and design priors, but also the specific properties of our utility function.

## Discussion

In this paper we investigated the potential of computationally optimizing experimental designs in associative learning studies, with the goal of enabling accurate statistical inference from experimental data. First, we formalized and parameterized the structure of classical conditioning experiments to render them amenable to design optimization. Next, we brought together several existing ideas from the literature on experimental design and computational optimization—in particular, simulation-based Bayesian experimental design and Bayesian optimization—and we composed them into a flexible method for designing associative learning studies. Finally, we evaluated the proposed method through simulations in three scenarios drawn from the literature on associative learning, with design optimization spanning both the goals of accurate parameter estimation and model selection. In these simulated scenarios, optimized designs outperformed manual designs previously used in similar contexts: the benefits ranged from a moderate reduction in parameter estimation error in Scenario 1, to substantial gains in model selection accuracies in Scenarios 2 and 3.

Furthermore, we highlight the role of prior knowledge in experimental design optimization. Within the framework of Bayesian experimental design, we can distinguish three types of prior probability distributions—the analysis, evaluation, and design prior. The analysis prior is the prior we ultimately plan to use in analyzing the obtained experimental data (if we plan to use Bayesian inference). Analysis priors should generally be acceptable to a wide audience, and therefore should not be overly informative or biased; for analysis priors we used flat priors that were fixed within scenarios, representing a fixed, but non-prejudiced analysis plan. The evaluation prior is used to simulate data for the evaluation of expected design utility: in simulations the models and their parameters are sampled from the evaluation prior. The design prior is used to simulate data in the process of optimizing experimental designs.

Although in practical applications the evaluation prior and the design prior coincide (both representing the designer’s partial knowledge), here we distinguish them in order to investigate how different levels of background knowledge impact the accuracy of designs under different assumed ground truths. Hence, in the presented scenarios, point evaluation priors represent the exactly assumed ground truth and design priors represent the varying degrees of background knowledge. For design priors we used both weakly-informative (vague) and strongly-informative priors. The weakly-informative and strongly-informative design priors can be thought of as providing approximate lower and upper bounds on achievable accuracy improvements, respectively. As expected, strongly-informative design priors provided highest accuracies, but, importantly, even the designs optimized with only weakly-informative design priors enabled similarly accurate inferences (e.g., in Scenarios 2 and 3). This result suggests that specifying the space of candidate models may in some cases be sufficient prior information to reap the benefits of design optimization, even when there is only sparse knowledge on plausible values of the models’ parameters.

### Limitations and future work

Although the results presented here show that optimized designs can yield substantial improvements over manual designs, there are some important caveats. For example, we can have floor or ceiling effects in design utility. A floor effect can be observed when, e.g., the data is noisy regardless of the chosen experimental design, leading to poor accuracy with both manual and optimized designs. Similarly, a ceiling effect can be observed when the utility of a manual design is already high—e.g., a design manually optimized for a small model space may already provide near-perfect model discrimination under low noise conditions. Nevertheless, given the often large number of design variables, and the complexity of the information that needs to be integrated to arrive at the optimal design, we conjecture that manual designs are often far from optimal. Support for this view comes from two recent studies. Balietti et al. (2018) [22] asked experts in economics and behavioral sciences to determine the values of two design variables of an economics game, such that the predictions of hypothesized models of behavior in this game would be maximally different. Results of simulations and empirical experiments showed that the experts suggested a more expensive and less informative experimental design, when comparing with a computationally optimized design. The study of Bakker et al. (2016) [47] investigated researchers’ intuitions about statistical power in psychological research: in an arguably simpler task of estimating statistical power of different designs, majority of the participants systematically over- or underestimated power. The findings of these two studies indicate the need for formal approaches.

Another important pair of caveats relates to the specification of the design optimization problem. First, the experiment can only be optimized with respect to the design variables that affect model responses. For example, in scenarios presented in this paper, we considered discrete, trial-level models of associative learning, rather than continuous-time models. However, trial-level models do not capture the effects of stimuli presentation timings, and therefore design variables related to timing cannot be optimized using this model space. If we suspect that the phenomenon under investigation crucially depends on timings, a model capturing this impact needs to be included in the model space. Second, if the specified models and their parameters inadequately describe the phenomenon of interest, then the optimized designs might actually perform worse than manually chosen designs. For example, if specified candidate models do not take into account attentional mechanisms or constraints on cognitive resources, the resulting optimized designs might either be so simple that they disengage subjects, or so complex that subjects cannot learn effectively. This issue can be solved directly by amending the candidate model space and design prior to better capture the studied phenomenon. Alternatively, the desired properties of the design can be achieved by including them in the utility function or by appropriately constraining design variables. Overall, it is important to keep in mind that design optimality is relative, not absolute: a design is only optimal with respect to the user-specified model space, design prior, experiment structure, design space, analysis procedure, and a utility function.

The problem of misspecification is however not particular to our approach, or even to experimental design in general; every statistical procedure requires assumptions, and can yield poor results when those assumptions are not met. However, in the domain of experimental design, this problem can be dealt with by iterating between design optimization, data acquisition, modeling, and model checking. Posterior of one iteration can be used as the design prior of the next one, and model spaces can be amended with new models, if current models are inadequate in accounting for the observed data. This iterative strategy is termed Adaptive Design Optimization (ADO) [48] and it can be implemented at different levels of granularity—between studies, between subsamples of subjects, between individual subjects, or even between trials. Implementing the ADO approach in the context of associative learning studies—especially with design updates at the level of each subject or trial—will be an important challenge for future work.

Adapting designs at finer granularity may be particularly beneficial for designs with complex experimental structures. Even global optimization algorithms like Bayesian optimization can struggle in high-dimensional experiment spaces. Optimizing designs on a per-trial basis may not only reduce the number of variables that need to be tuned simultaneously, but might also improve inference accuracy by incorporating new data as it becomes available [49]. But even with the non-adaptive design approach, a number of options is available to reduce the computational burden: Bayesian optimization can be sped up using GPUs [50], and analytical bounds [17] or normal approximations to the utility function [51] can be used to identify promising parts of the experiment space, which can then be searched more thoroughly (see Ryan et al. (2016) [52] for a review of computational strategies in Bayesian experimental design). Moreover, although some of the scenarios presented in this paper required lengthy optimizations, this time investment seems negligible compared to typical length of data acquisition in associative learning studies and to the cost of the study yielding non-informative data.

We have demonstrated our approach by optimizing designs for accurate single-subject inferences, which we contend is especially valuable from a translational perspective, and also represents a difficult benchmark, due to limited amounts of data in single-subject experiments. Group studies could also be accommodated within our approach, but simulating group studies would be computationally expensive, and it would imply that the design can be updated only once all the subjects’ data has been acquired. Instead, in future developments of design methods for associative learning, it may be advantageous to adopt the hierarchical ADO (HADO) approach [53]: this approach combines hierarchical Bayesian modeling with adaptive design. Even when the goal is to achieve accurate inferences at the level of single subjects, hierarchically combining the new subject’s data with the previously acquired data can improve efficiency and accuracy.

The design optimization method was illustrated on classical conditioning, as a special case of associative learning, but the extension to operant conditioning is conceptually straightforward. Whereas we formalized classical conditioning experiments using Markov chains, operant conditioning experiments can be formalized using Markov decision processes (MDPs). In general, optimizing MDPs may be computationally challenging, as the design has to specify transition probabilities and rewards for each state-action pair, possibly resulting in a high-dimensional problem. However, tractable optimization problems may also be obtained for experiments that can be represented using highly structured MDPs, with only few tunable design variables [20, 54]. We expect that the crucial challenge for future work will be to elucidate general correspondences between model classes and experimental structures that allow their discrimination, while still being amenable to optimization (i.e., resulting in low-dimensional problems).

### Conclusion

We believe that computational optimization of experimental designs is a valuable addition to the toolbox of associative learning science and that it holds promise of improving the accuracy and efficiency of basic research in this field. Optimizing experimental designs could also help address the recent concerns over underpowered studies in cognitive sciences [55]: the usually-prescribed use of larger samples should be complemented with the use of better designed experiments. Moreover, the effort in specifying the modeling details prior to designing the experiment allows for the experiment to easily be pre-registered, thus facilitating the adoption of emerging open science norms [56, 57]. The requirement to specify candidate models and the analysis procedures may seem restrictive, but this does not preclude subsequent exploratory analyses, it just delineates them from planned confirmatory analyses [58]. And the results of both confirmatory and exploratory analyses can then be used to further optimize future experiments.

Finally, we believe design optimization will play a role not only in basic research on associative learning, but also in translational research within the nascent field of computational psychiatry. Computational psychiatry strives to link mental disorders with computational models of neural function, or with particular parameter regimes of these models. Employing insights of computational psychiatry in a clinical setting will require the development of behavioral or physiological diagnostic tests, based on procedures like model selection and parameter estimation [59]. This presents a problem analogous to the design of experiments [60], and therefore computational optimization may be similarly useful in designing accurate and efficient diagnostic tests in this domain.

## Supporting information

S1 Appendix Formal approach to optimizing associative learning experiments. (PDF)
