# Supplementary material for: Computational optimization of associative learning experiments
Source: PLoS Comput Biol. 2020 Jan 6;16(1):e1007593. doi: 10.1371/journal.pcbi.1007593 (PMC6964915; doi:10.1371/journal.pcbi.1007593)
Supplement: S1 Appendix — (PDF) [file pcbi.1007593.s001.pdf]

S1 Appendix: Formal approach to optimizing associative learning experiments

Filip Melinscak* [1,2] and Dominik R. Bach[1,2,3]

[1]Computational Psychiatry Research; Department of Psychiatry, Psychotherapy, and Psychosomatics; Psychiatric Hospital; University of Zurich
[2]Neuroscience Center Zurich; University of Zurich
[3]Wellcome Centre for Human Neuroimaging and Max Planck/UCL Centre for Computational Psychiatry and Ageing Research; University College London

* Correspondence: Filip Melinscak <filip.melinscak@uzh.ch>

In this Appendix we first provide a technical treatment of simulation-based Bayesian experimental design. Next, we describe Bayesian optimization in the context of experimental design. Lastly, we give the full specification of the associative learning models used in simulated scenarios described in the main text of the article (Scenarios 1, 2, and 3).

1 Bayesian experimental design

Bayesian experimental design is the application of Bayesian decision theory to the problem of designing optimal experiments, i.e., finding the optimal values of tunable design variables. The problem is formalized as a maximization of *expected design utility* $EU(\eta)$, with respect to *design variables* η:

$$\eta^* = \underset{\eta \in H}{\arg\max}\, EU(\eta), \tag{1}$$

where η^* denotes optimal values for the design variables and H denotes the space of possible designs (i.e., *design space*). Although we are ultimately interested in the dependence of the experiment utility on the design variables (captured by the utility function $U(\eta)$), often it is easier to express the goal of the study by using a more general utility function with additional direct dependence on study outcomes (observed data d and analysis results r), and possibly on the unobserved ground truth generative model m and its parameters θ_m, yielding $U(r, d, \theta_m, m, \eta)$. Evaluating a particular design requires calculating expected design utility, i.e., marginalizing across the uncertain variables in the utility function (here we assume that analysis results r

are deterministically dependent on data d):

$$
\begin{aligned}
EU(\eta) &= \mathbb{E}_{d,\theta_m,m}[U(r,d,\theta_m,m,\eta)] \\
&= \int\limits_{\mathcal{D}} \sum_{\mathcal{M}} \int\limits_{\Theta_m} U(r,d,\theta_m,m,\eta) p(d,\theta_m,m \mid \eta) \, \mathrm{d}\theta_m \, \mathrm{d}d \\
&= \int\limits_{\mathcal{D}} \sum_{\mathcal{M}} \int\limits_{\Theta_m} U(r,d,\theta_m,m,\eta) p(d \mid \theta_m,m,\eta) p_e(\theta_m,m) \, \mathrm{d}\theta_m \, \mathrm{d}d,
\end{aligned}
\tag{2}
$$

where $\mathcal{D}$ is the space of datasets, $\mathcal{M}$ is the space of candidate models, Θ_m is the parameter space of model m, $p(d \mid \theta_m, m, \eta)$ is the conditional probability of the dataset, and $p_e(\theta_m, m)$ is the joint prior probability of the model and its parameters (which we term the *evaluation prior*). Here we made the assumption that the evaluation prior is independent of the design variables (i.e., $p_e(\theta_m, m \mid \eta) = p_e(\theta_m, m)$ for all η); this assumption needs to be carefully considered on a case-by-case basis, and might not hold if, for example, different values of design variables result in experiments which engage different cognitive mechanisms.

1.1 Evaluating design utility through simulation

Unfortunately, the expected design utility $EU(\eta)$, calculated according to eq. 2, will have no closed-form solution for almost any design problem of practical interest. To tackle this issue, we adopt the simulation-based approach to design evaluation, i.e., Monte Carlo integration (Müller and Parmigiani 1995; Wang and Gelfand 2002). Each simulation iteration consists of three steps: simulating a dataset, analyzing the simulated dataset, and calculating the utility of the simulated experiment.

To simulate a dataset, we first sample the ground truth model m and its parameters θ_m from the evaluation prior $p_e(\theta_m, m)$. Next, using the evaluated values of design variables η and the ground truth, we sample the dataset d from the data probability distribution $p(d \mid \theta_m, m, \eta)$. In classical conditioning, *experiment realizations* (i.e., stimuli and outcomes, together denoted x) are independent of *model responses* (i.e., conditioned responses, denoted y); hence, we can decompose the dataset simulation into sampling an experiment realization from the *experiment structure* $p_{\text{exp}}(x \mid \eta)$ and sampling the model responses from the *model likelihood function* $p_m(y \mid x, \theta_m)$, thus yielding the full simulated dataset $d = \{x, y\}$.

We can now perform the planned analysis on the simulated dataset. We conceptualize this with a functional mapping from the data to the results, i.e., $r = a(d)$, where $a(\cdot)$ represents the planned analysis (e.g., model comparison or parameter estimation) and r represents the analysis results (e.g., the selected winning model, the parameter point estimate, or a posterior distribution over parameters and/or models). In scenarios where we consider parameter estimation within a single model, we can obtain the *maximum a posteriori* (MAP) point estimate of the parameter as the analysis result r, using Bayes' rule:

$$
r = \hat{\theta}^{\text{MAP}} = \arg\max_{\theta} \frac{p(y \mid x, \theta) p_a(\theta)}{\int p(y \mid x, \theta) p_a(\theta) \, \mathrm{d}\theta}, \tag{3}
$$

where $p_a(\theta)$ is the *analysis prior*, which, importantly, does not need to coincide with the evaluation prior $p_e(\theta)$. Since the evaluation prior describes experimenter's state of knowledge at the planning stage, and the analysis prior needs to be acceptable even to the skeptical consumer of the analysis results, the evaluation prior will usually be more informative than the analysis prior. In scenarios presented in the paper, we used the uniform prior as a non-informative analysis prior $p_a(\theta) \propto 1$; this makes the MAP parameter estimate

equal to the maximum (log)likelihood estimate (MLE):

$$\hat{\theta}^{\text{MAP}} = \hat{\theta}^{\text{MLE}} = \arg\max_{\theta} \log p(y \mid x, \theta). \tag{4}$$

In model selection scenarios, we can similarly obtain the analysis result by employing the Bayes' rule to select the *a posteriori* most probable model, based on maximizing model evidence:

$$\begin{aligned} r = \hat{m} &= \arg\max_{m} p(m \mid y, x) \\ &= \arg\max_{m} p(y \mid x, m) \\ &= \arg\max_{m} \int p_m(y \mid x, \theta_m) p_a(\theta_m \mid m) \, \mathrm{d}\theta_m, \end{aligned} \tag{5}$$

where we assumed a uniform analysis prior over the models, i.e., $p_a(m) \propto 1$. Again, the integral necessary to calculate model evidence will generally not be tractable, but can be approximated in various ways (Penny et al. 2004).

Having obtained the analysis result from the simulated dataset, we can now calculate the experiment utility $U(r, d, \theta_m, m, \eta)$ of the current simulation iteration. In general, the utility function is dependent on the goal of the study and therefore its form is up to the user; here we provide two utility functions as examples - one for parameter estimation scenarios and one for model selection scenarios. In scenarios featuring parameter estimation within a single model, we decided to minimize the absolute parameter estimation error (i.e., maximizing negative absolute error), due to its robustness to outliers:

$$U(r = \hat{\theta}, \theta) = -|\theta - \hat{\theta}|. \tag{6}$$

Similarly, in scenarios featuring model selection, we decided to maximize model selection accuracy:

$$U(r = \hat{m}, m) = \begin{cases} 1 & \text{if } \hat{m} = m, \\ 0 & \text{otherwise.} \end{cases} \tag{7}$$

Thus far, we have described calculating the experiment utility for a single simulation. To obtain the Monte Carlo approximation of the expected design utility $EU(\eta)$ (eq. 2), we run N_S simulations and average experiment utilities across them:

$$EU(\eta) \approx \frac{1}{N_S} \sum_{i=1}^{N_S} U(r^{(i)}, d^{(i)}, \theta_m^{(i)}, m^{(i)}, \eta). \tag{8}$$

1.2 Maximizing design utility through Bayesian optimization

Using the Monte Carlo approximation to the expected design utility (eq. 8), we can now search the design space H for the optimal design η^* that maximizes expected utility $EU(\eta)$ (eq. 1). We refer to the evaluation prior $p_e(\theta_m, m)$ used during optimization as the *design prior* $p_d(\theta_m, m)$. In practice, the design prior and the evaluation prior will generally coincide, since we want to use the best existing information to design the experiment and to evaluate it. However, the distinction between the design prior and the evaluation prior allows us to investigate the effect of varying levels of prior knowledge included in the optimization (i.e.,

different design priors), while evaluating the obtained designs using the same evaluation prior (which here represents the ground truth and will usually be a point prior).

To find the values of design variables that maximize expected design utility, we employ the Bayesian optimization (BO) algorithm (Brochu, Cora, and de Freitas 2010), as implemented by the `bayesopt` function in the 'Statistics and Machine Learning Toolbox' in MATLAB R2018a (version 9.4.0.813654). Since optimization algorithms are usually formulated in terms of minimizing an objective function $g(x)$, we equate the objective function with negative expected design utility, i.e., $g(x) = -EU(\eta)$. BO is a global optimization algorithm which minimizes a deterministic or stochastic objective function $g(x)$, with respect to x within a bounded domain. BO is especially appropriate for optimization problems where the objective function is expensive to evaluate and where the gradients of the objective function are not available; both of these conditions are true for simulation-based experimental design. Although the BO algorithm has polynomial time complexity, for expensive objective functions and for typical numbers of iterations, the running time will be dominated by the evaluations of the objective function, thus justifying the use of a relatively costly search algorithm.

In order to efficiently search for the optimal x, BO approximates the true objective function $g(x)$ using a probabilistic model $f(x)$, called the *surrogate function* (or *response surface*). A common choice for the surrogate function (also featured in the MATLAB BO implementation) is a Gaussian process:

$$f(x) \sim \mathcal{GP}(\mu(x), k(x, x'; \theta)), \tag{9}$$

where $\mu(x)$ is the mean function and $k(x, x'; \theta)$ is the covariance kernel function with hyperparameters θ. BO implementation in MATLAB uses the ARD Matern 5/2 form of the kernel function. In order to choose the next solution x to evaluate, the surrogate function needs to be supplemented with an *acquisition function* $u(x)$, which governs the exploration-exploitation trade-off. The acquisition function represents the utility of evaluating a solution x, based on the current posterior probability distribution over the objective function, $Q(f)$. Out of various acquisition functions that are available in the MATLAB implementation of BO, we have chosen the '*expected improvement plus*' option. The expected improvement acquisition functions evaluate the expected decrease in the objective function, ignoring values that cause an increase in the objective. Expected improvement (EI) is given by:

$$EI(x, Q) = \mathbb{E}_Q[\max(0, \mu_Q(x_{\text{best}}) - f(x))], \tag{10}$$

where x_{best} is the solution with the lowest posterior mean value $\mu_Q(x_{\text{best}})$. The EI-plus version of the acquisition function dynamically modifies the kernel function to prevent overexploiting of a particular area of the solution space. This modification is governed by the 'exploration ratio' option, which was set to the default value of 0.5.

Having specified the surrogate and acquisition functions, the BO algorithm proceeds as follows. The algorithm is first initialized by evaluating $y_i = g(x_i)$ for a fixed number of solutions x_i sampled randomly within variable bounds (i indexes objective function evaluations); we used 10 solutions in initialization. Hereafter, the algorithm repeats the following steps in each iteration t:

1. Update the surrogate function using the Bayes' rule, i.e., calculate the posterior over functions, conditioned on the objective values observed thus far ($Q(f \mid x_i, y_i, \text{for } i = 1, \ldots, t)$).
2. Find the new solution x_{t+1} that maximizes the acquisition function $u(x)$ and evaluate the objective

function for this solution to obtain $y_{t+1} = g(x_{t+1})$.

As the stopping criterion for the algorithm, we used a fixed number of iterations (300), regardless of the elapsed time. The BO algorithm returns both the solution at which the lowest objective function value was observed, as well as the solution at which the final surrogate function has the lowest mean. As the optimized design, we chose the solution based on the surrogate function.

2 Associative learning models

2.1 Scenario 1

In Scenario 1 we consider the case of estimating the learning rate parameter of the Rescorla-Wagner (RW) model (Rescorla and Wagner 1972). For this model, the update rule for the associative weights between the stimuli s and outcome o is:

$$\mathbf{w}_{t+1} = \mathbf{w}_t + \alpha\delta_t\mathbf{s}_t, \tag{11}$$

where $\mathbf{w}_t$ is the vector of associative weights at the end of trial t, α is the learning rate, δ_t is the prediction error, and $\mathbf{s}_t$ is the vector of binary indicator variables for the stimuli (i.e., conditioned stimuli, CSs). The prediction error is given by:

$$\delta_t = o_t - \mathbf{w}_t^\top\mathbf{s}_t, \tag{12}$$

where o_t is the binary indicator variable for the outcome (i.e., unconditioned stimulus, US). The mapping from the associative weights $\mathbf{w}_t$, to the model responses y_t (i.e., conditioned responses, CRs) is assumed to be linear with added Gaussian noise:

$$\begin{aligned} y_t &= \beta_0 + \beta_1\mathbf{s}_t^\top\mathbf{w}_t + \epsilon, \\ \epsilon &\overset{i.i.d.}{\sim} \mathcal{N}(0, \sigma_y^2). \end{aligned} \tag{13}$$

The parameters of the model are therefore α, $\mathbf{w}_0$, β_0, β_1, and σ_y. We make the simplifying assumption that all initial values of associative weights are the same ($\mathbf{w}_0 = w_0\mathbf{1}$). In all the simulations, parameters other than α were kept constant ($w_0 = 0$, $\beta_0 = 0$, $\beta_1 = 1$, $\sigma_y = 0.2$). The parameters were estimated using the maximum likelihood approach, which is equivalent to the maximum a posteriori approach with uniform priors on the whole domain of permissible values for the parameter (eq. 4). To perform the estimation we used the `mfit_optimize` function from the 'mfit' toolbox (Gershman 2018).

Three evaluation priors, with different values for α were used: low alpha (LA, $\alpha = 0.1$), middle alpha (MA, $\alpha = 0.2$), and high alpha (HA, $\alpha = 0.3$). The designs were optimized using either a point prior (PA-OPT designs) or a vague prior (VA-OPT designs) on alpha. The PA-OPT design priors coincided with evaluation priors, and the VA-OPT design prior was a uniform distribution on the [0, 1] interval. The designs were optimized for accuracy in estimating the learning rate α, with the utility function being specified as the negative absolute estimation error (eq. 6).

2.2 Scenario 2

Scenario 2 involves model comparison between the RW model and its probabilistic extension, the Kalman RW (KRW) model (Dayan and Kakade 2000; Kruschke 2008; Gershman 2015). The RW model is implemented the same as in Scenario 1 (equations 11–13). KRW model maintains a probabilistic representation of associative weights using a multivariate Gaussian distribution, with mean vector $\mathbf{w}_t$ and covariance matrix $\mathbf{\Sigma}_t$. These

state variables are updated using the Kalman filter equations:

$$\mathbf{w}_{t+1} = \mathbf{w}_t + \mathbf{k}_t \delta_t, \tag{14}$$

$$\mathbf{\Sigma}_{t+1} = \mathbf{\Sigma}_t + \tau^2 \mathbf{I} - \mathbf{k}_t \mathbf{s}_t^\top (\mathbf{\Sigma}_t + \tau^2 \mathbf{I}), \tag{15}$$

where τ^2 is the variance that governs the diffusion of weights over time, and $\mathbf{k}_t$ is the Kalman gain, which acts as a stimulus-specific learning rate. Kalman gain is given by:

$$\mathbf{k}_t = \frac{(\mathbf{\Sigma}_t + \tau^2 \mathbf{I})\mathbf{s}_t}{\mathbf{s}_t^\top (\mathbf{\Sigma}_t + \tau^2 \mathbf{I})\mathbf{s}_t + \sigma_o^2}, \tag{16}$$

where σ_o^2 is the variance of the outcome noise distribution. The model responses y_t for KRW are obtained as a linear mapping of associative weights $\mathbf{w}_t$ (eq. 13). Hence, the parameters of the model are $\mathbf{w}_0$, $\mathbf{\Sigma}_0$, β_0, β_1, τ, σ_o, and σ_y. We simplify the parameterization by assuming $\mathbf{w}_0 = w_0 \mathbf{1}$ and $\mathbf{\Sigma}_0 = \sigma_w^2 \mathbf{I}$.

The evaluation prior was a uniform prior over RW and KRW models, combined with a point prior on model parameters, with the parameter values taken from the simulation study of Kruschke (2008). For RW, parameter values were $w_0 = 0$ and $\alpha = 0.08$. For KRW, we set the parameters at $w_0 = 0$, $\log \sigma_w^2 = 0$ ($\sigma_w^2 = 1$), $\log \tau^2 = -20$ ($\tau^2 \approx 0$), $\log \sigma_o^2 = 1.3863$ ($\sigma_o^2 \approx 4$). For both models, the parameters of the model response mapping were set at $\beta_0 = 0$, $\beta_1 = 1$, and $\sigma_y = 0.2$.

The design prior was either a precise point prior coinciding with the evaluation prior (PP-OPT), or a vague prior (VP-OPT). The vague prior for the RW model was implemented as:

$$\begin{aligned} \alpha &\sim \mathcal{U}(0, 1), \\ \sigma_y &\sim \mathcal{U}(0.05, 0.5). \end{aligned} \tag{17}$$

For the KRW model, the vague prior was:

$$\begin{aligned} \log \sigma_w^2 &\sim \mathcal{N}(0, 2), \\ \log \sigma_o^2 &\sim \mathcal{N}(0, 2), \\ \sigma_y &\sim \mathcal{U}(0.05, 0.5). \end{aligned} \tag{18}$$

The rest of the parameters of both models were fixed to the same values as in the evaluation prior.

Parameter estimates in Scenario 2 were obtained using a maximum likelihood approach, as in Scenario 1. Models were compared using the Bayesian Information Criterion (BIC; (Schwarz 1978)):

$$\text{BIC} = -2 \log(\hat{L}) + \log(n) k, \tag{19}$$

where $\hat{L}$ is the value of the likelihood function for the maximum likelihood parameter estimates, n is the number of data points, and k is the number of estimated parameters. The model with the lowest BIC was selected as the winning model. When conducting design optimization, proportion of cases where the winning model was also the ground truth model - i.e., model selection accuracy - was transformed to the log-odds scale in order to better conform with the normality assumption we used in optimization (we assumed a Gaussian process as a surrogate model of the utility function in Bayesian optimization).

2.3 Scenario 3

Scenario 3 again features model comparison between the RW model and its variant - namely, the Rescorla-Wagner-Pearce-Hall (RWPH) hybrid model (Li et al. 2011). Whereas the RW model assumes a fixed learning rate, the RWPH model assumes stimulus-specific learning rate that is modulated over time depending on observed prediction errors. In particular, the RW weight updating is supplemented with the Pearce-Hall rule for updating the learning rates (i.e., associabilities):

$$\alpha_{t+1}^{(s)} = \begin{cases} \eta|\delta_t| + (1-\eta)\alpha_t^{(s)} & \text{if } s_t = 1, \\ \alpha_t^{(s)} & \text{if } s_t = 0, \end{cases} \tag{20}$$

where $\alpha_t^{(s)}$ is the associability of stimulus s at trial t and η is the forgetting factor. The corresponding weight update equation is:

$$w_{t+1}^{(s)} = w_t^{(s)} + \kappa\alpha_t^{(s)}\delta_t s_t, \tag{21}$$

where κ is the fixed learning rate. Additionally, in Scenario 3 we included variants of the RWPH model that have different mappings between the latent states of the models (i.e., weights and associabilities) and the model responses (i.e., conditioned responses). The most general version of the model, RWPH($V+\alpha$), features a linear mapping from a mixture of active weights and associabilities:

$$y_t = \beta_0 + \beta_1 \mathbf{s}_t^\top(\lambda\mathbf{w}_t + (1-\lambda)\boldsymbol{\alpha}_t) + \epsilon, \tag{22}$$

where λ is the mixing factor. The parameters of the model are $\boldsymbol{\alpha}_0$, $\mathbf{w}_0$, η, β_0, β_1, λ, and σ_y. Again, we make the simplifying assumption about the initial associabilities and weights (all initial associabilities and weights are equal to α_0 and w_0, respectively). The other variations of the RWPH model - RWPH(V) and RWPH(α) - are obtained by setting the output mixing factor λ to 1 or 0, respectively.

As in Scenario 2, the evaluation prior was a uniform prior over all compared models (RW, RWPH(V), RWPH(α), RWPH($V+\alpha$)), combined with a point prior on model parameters. The parameters of RWPH($V+\alpha$) update equations were set to the maximum likelihood values obtained in the empirical study of Li et al. (2011); specifically, $w_0 = 0$, $\alpha_0 = 0.926$, $\eta = 0.166$, and $\kappa = 0.857$. For the other models - which are nested within the RWPH($V+\alpha$) model - corresponding subsets of these maximum likelihood values were used. Study of Li et al. (2011) did not specify the maximum likelihood values of output mapping parameters, so they were arbitrarily set at $\beta_0 = 0$, $\beta_1 = 1$, and $\sigma_y = 0.2$. The mixing factor λ was set to 0.5 in RWPH($V+\alpha$), to 0 in RWPH(α), and to 1 in RWPH(V) and RW.

The design prior - as in Scenario 2 - was either a point prior coinciding with the evaluation prior (PP-OPT), or a vague prior (VP-OPT). The vague prior for the RWPH($V+\alpha$) model was:

$$\begin{aligned} \alpha_0 &\sim \mathcal{U}(0,1), \\ \eta &\sim \mathcal{U}(0,1), \\ \kappa &\sim \mathcal{U}(0,1), \\ \lambda &\sim \mathcal{U}(0,1), \\ \sigma_y &\sim \mathcal{U}(0.05, 0.5). \end{aligned} \tag{23}$$

For the other models we used the appropriate subset of priors. The rest of the parameters of all the mod-

els were fixed to the same values as in the evaluation prior. Model fitting, model selection, and design optimization were performed in the same manner as in Scenario 2.

3 References

Brochu, Eric, Vlad M. Cora, and Nando de Freitas. 2010. "A Tutorial on Bayesian Optimization of Expensive Cost Functions, with Application to Active User Modeling and Hierarchical Reinforcement Learning." *arXiv:1012.2599 [Cs]*, December. http://arxiv.org/abs/1012.2599.

Dayan, Peter, and Sham Kakade. 2000. "Explaining Away in Weight Space." In *Proceedings of the 13th International Conference on Neural Information Processing Systems*, 430–36. NIPS'00. Cambridge, MA, USA: MIT Press.

Gershman, Samuel J. 2018. "'Mfit': Simple Model-Fitting Tools." *GitHub Repository.* https://github.com/sjgershm/mfit.

———. 2015. "A Unifying Probabilistic View of Associative Learning." Edited by Jörn Diedrichsen. *PLOS Computational Biology* 11 (11): e1004567. https://doi.org/10.1371/journal.pcbi.1004567.

Kruschke, J. K. 2008. "Bayesian Approaches to Associative Learning: From Passive to Active Learning." *Learning & Behavior* 36 (3): 210–26. https://doi.org/10.3758/LB.36.3.210.

Li, Jian, Daniela Schiller, Geoffrey Schoenbaum, Elizabeth A Phelps, and Nathaniel D Daw. 2011. "Differential Roles of Human Striatum and Amygdala in Associative Learning." *Nature Neuroscience* 14 (10): 1250–2. https://doi.org/10.1038/nn.2904.

Müller, Peter, and Giovanni Parmigiani. 1995. "Optimal Design via Curve Fitting of Monte Carlo Experiments." *Journal of the American Statistical Association* 90 (432): 1322–30. https://doi.org/10.1080/01621459.1995.10476636.

Penny, W. D., K. E. Stephan, A. Mechelli, and K. J. Friston. 2004. "Comparing Dynamic Causal Models." *NeuroImage* 22 (3): 1157–72. https://doi.org/10.1016/j.neuroimage.2004.03.026.

Rescorla, Robert A, and Allan R Wagner. 1972. "A Theory of Pavlovian Conditioning: Variations in the Effectiveness of Reinforcement and Nonreinforcement." In *Classical Conditioning II Current Research and Theory*, 21:64–99. New York: Appleton-Century-Crofts.

Schwarz, Gideon. 1978. "Estimating the Dimension of a Model." *The Annals of Statistics* 6 (2): 461–64. https://doi.org/10.1214/aos/1176344136.

Wang, Fei, and Alan E. Gelfand. 2002. "A Simulation-Based Approach to Bayesian Sample Size Determination for Performance Under a Given Model and for Separating Models." *Statistical Science* 17 (2): 193–208. https://doi.org/10.1214/ss/1030550861.
